# Sustained high glucose intake accelerates type 1 diabetes in NOD mice

**DOI:** 10.3389/fendo.2022.1037822

**Published:** 2022-12-05

**Authors:** Xiangqian Li, Lina Wang, Gang Meng, Xiaoling Chen, Shushu Yang, Mengjun Zhang, Zhengni Zheng, Jie Zhou, Zhu Lan, Yuzhang Wu, Li Wang

**Affiliations:** ^1^ Institute of Immunology People's Liberation Army (PLA) & Department of Immunology, College of Basic Medicine, Army Medical University (Third Military Medical University), Chongqing, China; ^2^ Department of Immunology, College of Basic Medicine, Qingdao University. Qingdao, Shandong, China; ^3^ Department of Immunology, College of Basic Medicine, Weifang Medical University, Weifang, China; ^4^ Department of Pathology, Southwest Hospital, Army Medical University (Third Military Medical University), Chongqing, China; ^5^ Department of Pharmaceutical Analysis, College of Pharmacy, Army Medical University (Third Military Medical University), Chongqing, China; ^6^ Department of Dermatology, Southwest Hospital, Army Medical University (Third Military Medical University), Chongqing, China

**Keywords:** type 1 diabetes, high glucose, proteomics, RNAseq, endoplasmic reticulum stress, dendritic cells

## Abstract

**Introduction:**

Epidemiological studies have suggested that dietary factors, especially high consumption of high glycaemic index carbohydrates and sugars, may trigger or exacerbate the progression of type 1 diabetes. We aimed to provide experimental evidence to confirm this relevance and to explore the underlying mechanisms.

**Methods:**

NOD mice were given sustained high-glucose drinking or glucose-free water and observed for the incidence of type 1 diabetes and islet inflammation. RNAseq was performed to detect the transcriptome changes of the NOD islet beta cell line NIT-1 after high glucose treatment, and mass spectrometry was performed to detect the proteome changes of NIT-1-cells-derived sEVs.

**Results:**

Sustained high glucose drinking significantly aggravates islet inflammation and accelerates the onset of type 1 diabetes in NOD mice. Mechanistically, high glucose treatment induces aberrant ER stress and up-regulates the expression of autoantigens in islet beta cell. Moreover, high glucose treatment alters the proteome of beta-cells-derived sEVs, and significantly enhances the ability of sEVs to promote DC maturation and stimulate immune inflammatory response.

**Discussion:**

This study provides evidence for negative effect of high glucose intake as a dietary factor on the pathogenesis of type 1 diabetes in genetically predisposed individuals. Therefore, avoiding high sugar intake may be an effective disease prevention strategy for children or adults susceptible to type 1 diabetes.

## Introduction

Type 1 diabetes (T1D) is an organ-specific autoimmune disease characterized by insulin deficiency, hyperglycemia, and complications due to inappropriate treatment (1). Worldwide, the incidence of T1D increased 3–4% annually over the past 30 years, and T1D develops more in children and adolescents than adults, resulting in a heavy social and medical burden (2–4). The rise in the incidence of T1D cannot be attributed to genetic factors alone; environmental factors may also contribute (5, 6). So far, the exact pathogenesis of T1D is not completely clear. Increasing evidence indicates that endoplasmic reticulum stress (ERS) in beta cells may lead to the generation and exposure of autoantigens and the activation of autoreactive T cells, and eventually result in the destruction of beta cells (7–9). Drugs that inhibit ERS can alleviate the incidence of T1D (10). Small extracellular vesicles (sEVs, 30-150 nm) are thought to be autoantigen carriers with potent adjuvant activity and may act as autoimmune triggers in T1D (11).

Adverse circumstances such as viral infection, inflammation, diet, stress, and toxins can lead to ERS in beta cells and initiate T1D development. Viral infection, as potential triggers for the pathogenesis of T1D, has been well studied (12, 13). Diet is another possible risk factor involved in the development of T1D, which has received increasing attention in recent years (14, 15). The consumption of sugar-sweetened beverages (SSBs) is increasing every year around the world, especially among children, which leads to a series of diseases, including obesity, dental caries and diabetes (16). In the mid-1990s, children’s intake of sugared beverages passed that of milk (17). Some studies have reported that consumption of sugar, specifically SSBs is widely considered the cause of the global rise in type 2 diabetes (T2D) (18, 19). Although an epidemiological survey showed that the possibility of islet autoimmunity progression to T1D in children was significantly correlated with total sugar intake (20), there is still lack of laboratory evidence that high-glucose intake promotes the pathogenesis of T1D. And it is not clear whether beta cells, in responding to high glucose-induced ERS, may stimulate an enhanced autoimmune response *via* altered sEVs.

In this study, we demonstrate that sustained high-glucose drinking significantly accelerates the onset of T1D in NOD mice. We found that high-glucose treatment can significantly enhance ERS and autoantigens expression in NIT-1 cell, a NOD mouse pancreatic beta-cell line. Additionally, high-glucose treatment can significantly enhance the immunostimulatory ability of beta-cell-derived sEVs. Mass spectrometry analysis shows that high-glucose treatment can significantly change cargo of NIT-1-cells-derived sEVs, and increase their ability to promote maturation of dendritic cells (DCs). We propose that a high-glucose diet is an important dietary factor in the development of T1D in genetically predisposed individuals.

## Materials and methods

### Mice treatment

NOD/Ltj mice were purchased from Beijing Huafukang Biotechnology Co, Ltd. All mice were bred and maintained in specific pathogen-free facilities and handled according to “Principles of Laboratory Animal Care and Use in Research” (Ministry of Health, China). NOD/Ltj mice were allowed to freely intake 1.1M glucose water starting at 4 weeks of age until onset of T1D. Control NOD/Ltj mice were given normal water. The intake of food and water in each cage of mice were recorded every week. Each NOD mouse given high-glucose drinking consumed about 1g glucose per day from water, and thus consumed nearly 50% less food than NOD mice given normal water. Random blood glucose was monitored using a glucometer (OneTouch Ultral, LifeScan). Once random blood glucose exceeded 13.9 mM for two consecutive days, NOD mice were diagnosed as T1D and euthanized immediately. All experimental protocols were approved by the Animal Ethics Committee of the Army Medical University (Third Military Medical University).

### Isolation and identification of sEVs

We isolated sEVs from cell culture supernatants according to MISEV guidelines (PMID: 30637094). NIT-1 cells were cultured within passages 20 (the cell culture time < one month) to ensure the stability of the cells. To prepare control sEVs, NIT-1 cells were cultured in 75-cm^2^ culture flasks using Ham’s F-12 Nutrient Mixture containing 7mM glucose (Thermo Fisher Scientific, USA), supplied with 10% exosomes-free FCS (icell, China) for 48 hours, and the cell viability was strictly guaranteed to be above 95% during cell culture. For high-glucose (HG) treatment, NIT-1 cells were pretreated with 20mM glucose for 24 hours. After the supernatants of cell culture were collected, the sEVs were isolated by ultracentrifugation. Briefly, the supernatants were centrifuged at 300g for 5 min to discard dead cells, then 2000g for 30 min, and 12,000g for 45 min at 4°C, followed by filtration using 450-nm pore-size membrane to remove cell debris and large particles. The sEVs were collected by spinning the final supernatants in a himac ultracentrifuge (Koki Holding Co. Ltd, Japan) at 110,000g for 70 min with P40ST-2690 rotor. Braking degree is set to 3 of 9. After washing once with an equal volume of PBS, the sEVs pellet was resuspended in PBS (1:500 concentrated from the original volume of culture supernatant). Protein concentration was determined by BCA Protein Assay Kit (Beyotime, China) and record the volume and particle concentration of the supernatant every time. Then the sEVs were identified by particle size detection, electron microscopy and western blotting. The size and concentration of particles were detected by NTA (nanoparticle tracking analysis).

### Isolation of bone marrow-derived dendritic cells and co-culture with sEVs

Bone marrow cells were obtained from the femurs and tibias of NOD/Ltj mice, and red blood cells were eliminated using ACK Lysis Buffer (Beyotime, China). Then bone marrow cells were washed and cultured in six-well tissue culture plates at 1×10^6^ cells/mL in complete RPMI culture medium supplemented with 10% fetal bovine serum and GM-CSF (20 ng/ml, Abcam) at 37°C, 5% CO2. The culture medium was changed on culture day 2 and 4. Fresh medium and GM-CSF were added after flushing out non-adherent cells. On day 6, loosely adherent clustered cells were harvested as immature BMDCs. To detect the ability of sEVs to promote DC maturation, immature BMDCs, cultured in complete medium supplemented with GM-CSF (10ng/ml) and IL-4 (5ng/ml), were treated with HG-sEV and C-sEV (9×10^9^ particles/ml) respectively for 24 h. LPS (1μg/ml) was used as a positive stimulant to promote DC maturation.

### Isolation of splenocytes and CD11c^+^ cells depletion

The spleen tissues were taken out from NOD/Ltj mice, and mashed on a sterile nylon mesh to prepare splenocytes suspension. Then splenocytes were re-suspended with ACK Lysis Buffer (Beyotime, China) for 2 min after centrifugation, and finally washed twice with PBS buffer. CD11c^+^ cells were depleted from splenocytes using the EasySep™ Mouse CD11c Positive Selection Kit II (#18780) according to the manufacturer’s instruction. Briefly, 0.5 ml splenocytes suspension at 1×10^8^ cells/ml was added into 5 ml (12×75 mm) polystyrene round-bottom tube, and 50 µl/ml rat serum was added. Then 25 µl selection cocktail (12.5 µl of Component A + 12.5 µl of Component B) was added into it and incubated for 5 min at room temperature. After incubating the cell sample with 20 µl RapidSpheres™ for 3 min, the tube was filled with about 2 ml medium and placed into the magnet. Finally, supernatant which contained splenocytes without CD11c^+^ cells was poured out. The purity of CD11c^-^ splenocytes was detected by flow cytometry.

### Proteome bioinformatics analysis

The three batches of beta-cell-derived sEVs from two groups were prepared. This project used the high-resolution mass spectrometer to analysis samples. Quantitative analysis was performed based on information such as peak intensity, peak area, and LC retention time of peptides related to first-order mass spectrometry. Briefly, proteins were extracted and digested into peptides by Trypsin enzyme, then peptide liquid was freeze-dried after salt removal. The dried peptide samples were reconstituted with mobile phase A (2% ACN, 0.1% FA), centrifuged at 20,000g for 10 minutes, and the supernatant was taken for injection. Separation was performed by Thermo UltiMate 3000 UHPLC. The nanoliter liquid phase separation end was directly connected to the mass spectrometer. The peptides separated by liquid phase chromatography were ionized by a nano-ESI source and then passed to a tandem mass spectrometer Orbitrap Fusion Lumos (Thermo Fisher Scientific, San Jose, CA) for DDA (Data Dependent Acquisition) mode detection. The main parameters were set: ion source voltage was set to 2kV, MS1 mass spectrometer scanning range was 350~1,500m/z; resolution was set to 60,000; MS2 starting m/z was fixed at 100; resolution was 15,000. The ion screening conditions for MS2 fragmentation: charge 2+ to 6+, and the top 30 parent ions with the peak intensity exceeding 20,000. The ion fragmentation mode was HCD, and the fragment ions were detected in Orbitrap. The dynamic exclusion time was set to 30 seconds. The AGC was set to: MS1 1E5, MS2 2E4.

The MS data were identified using the MaxQuant’s integrated Andromeda engine. UniProtKB/Swiss-Prot subset were used for protein identification. At the spectrum level, filtering was performed with PSM-level FDR ≤ 1%, and at the protein level, further filtering was performed with Protein-level FDR ≤ 1% to obtain significant identification result.

### RNA-seq and analysis of differentially expressed genes

NIT-1 cells were cultured in 75-cm^2^ culture flasks using Ham’s F-12 Nutrient Mixture containing 7 mM glucose. For high-glucose (HG) treatment, NIT-1 cells were pretreated with 20mM glucose for 24 hours. Cells from two groups were collected for RNA-Seq. After enriching mRNA with Oligo (dT) magnetic beads, mRNA was fragmented by breaking reagent, and the first and second strands of cDNA were synthesized by PCR instrument. After the end repair and joint connection, the PCR reaction and product recovery were carried out. Finally, the final library was obtained by cyclization of the product. Agilent 2100 Bioanalyzer was used to detect the fragment size and concentration of the library. Finally, the sequence reading length of 50bp/100bp/150bp was obtained by combined probe anchored polymerization.

The sequenced raw reads were filtered by SOAPnuke software (21) to remove joint contamination, reads with unknown base N content greater than 5% and low-quality reads. HISAT (22) was used to compare the clean reads obtained by quality control with the reference genome sequence of mouse genome, and the gene quantitative analysis was carried out. The screening of differential genes was based on DEseq2 method (23), which was negative binomial distribution, and the differentially expressed genes (DEGs) between HG group and C group were screened. FDR< 0.05 and |fold change, FC| ≥ 1.5 were set as filter thresholds for DEGs identification. Pearson correlation analysis of RNA-seq data indicated that one of the three biological replicates in the control group deviated greatly from the other two, so we removed the one with lower correlation when analyzing the data. The Dr. Tom platform independently developed by BGI was used to carry out more in-depth analysis of DEGs, such as cluster analysis, Gene Ontology (GO) and Kyoto Encyclopedia of Genes and Genomes (KEGG) enrichment analysis.

### Mouse IFN-Υ ELISPOT Assays

The ability of NIT-1 cells or their sEVs to stimulate IFN-γ secretion by splenocytes of NOD mice was evaluated using an ELISPOT assay. Briefly, 10^5^ splenocytes or CD11c^+^ depleted splenocytes from 10-week-old NOD mice were inoculated in each well of anti-mouse IFN-γ antibody-precoated ELISPOT plates, and co-cultured with 10^4^ high glucose-treated or untreated NIT-1 cells or their sEVs (9×10^9^ particles/ml) for 24 hours, respectively. Splenocytes cultured alone were used for negative control, and splenocytes stimulated with PMA and ionomycin were used for positive control. After incubation, the cells were removed and plates were processed according to the instructions of mouse IFN-γ precoated ELISPOT kit (Cat#: DKW22-2000-096). The spots were counted using a spot reader system (Saizhi, Beijing, China).

### Real-time quantitative PCR

Total RNA was isolated with RNAiso Plus (Takara, Japan) from NIT-1 cells of two groups and cDNA was synthesized with PrimeScript™ RT reagent Kit with gDNA Eraser (Perfect Real Time) (Takara, Japan). Quantification of cDNA was performed by qRT-PCR (iCycler, BioRad, USA) with the TB Green Premix Ex Taq™ ll (Tli Rnaseh Plus) (Takara, Japan). Cycling parameters were 95°Cfor 3 min and 39 cycles of 95°C for 10 s, 58 °C for 10 s, and 72 °C for 20 s. The sequences used were as follows: mouse Atf6, Sense 5’-TCGCCTTTTAGTCCGGTTCTT-3’, Anti-sense 5’-GGCTCCATAGGTCTGACTCC-3’; mouse eIF-2α, Sense 5’-ATGCCGGGGCTAAGTTGTAGA-3’; Anti-sense 5’-AACGGATACGTCGTCTGGATA-3’; mouse Chga, Sense 5’-CCAAGGTGATGAAGTGCGTC-3’; Anti-sense 5’-GGTGTCGCAGGATAGAGAGGA-3’; mouse Txnip, Sense 5’-GGCCGGACGGGTAATAGTG-3’; Anti-sense 5’-AGCGCAAGTAGTCCAAAGTCT-3’; mouse Xbp-1s Sense 5’-CTGAGTCCGAATCAGGTGCAG-3’; Anti-sense 5’-GTCCATGGGAAGATGTTCTGG-3’;

Gene expression was normalized using the △△C_t_ method, where the amount of target, normalized to an endogenous reference and relative to a calibrator, is given by 2^−△△Ct^, where C_t_ is the cycle number of the detection threshold.

### Flow cytometry

Single-cell suspensions were made from spleens and pancreas infiltrating immune cells of 12-15-week-old NOD mice. Fluorochrome-conjugated antibodies specific for surface markers used in this study were anti-CD3-PE (145-2C11), anti-CD4-PE-Cy™7 (RM4-5), anti-CD8-BV510 (53-6.7), anti-CD11c-FITC (N418), anti-(I-A/I-E)-PE-Cyanine7 (M5/114.15.2), Anti-CD86-APC (GL-1) (eBiosciences, USA); and anti-IFN-γ-APC (XMG1.2) (eBiosciences, USA) for Intracellular staining. All samples were stained with Fixable Viability Dye eFluor™ 780 (Invitrogen™, USA), to facilitate live-cell gating before cell surface and intracellular staining. Doublets were excluded with forward scatter height against forward scatter area and subsequently side scatter height against side scatter area. All data were acquired on an BD FACSCanto™ II Flow Cytometer (Biosciences, USA) and analyzed with FlowJo software (Tree Star, Inc, USA).

### IA-2 antibody ELISA

The mouse IA-2 antibody (IgG) ELISA was performed according to the manufacturer’s suggested protocol. Briefly, serums from mice fed with high-glucose water and normal water were run in duplicate to determine IA-2 antibody titers. Optical densities were determined with a microplate reader (800 TS, Biotek) set to 450 nm, with the correction wavelength set to 540 nm.

### Histopathology

At the end of the experimental procedure, mice were dissected by cervical dislocation and mice pancreas and spleen quickly removed. Mice pancreas after fixation with 4% formaldehyde, were processed for histopathological examinations using hematoxylin and eosin staining due to instructions of a standard protocol. Each mouse included at least 10 islets. Insulitis was scored as below: 0, no insulitis; 1, peri-insulitis; 2, mild insulitis (<25% of infiltrated islets); 3, severe insulitis (25–75% of infiltrated islets).

### SDS-PAGE and western blotting analysis

The sEVs lysates were prepared by resuspending sEVs pellets in equal volume sEV-lysing buffer (Umibio, China), and then incubated on ice for 10 min, followed by centrifugation at 12,000 rpm for 5 min at 4˚C. To perform SDS-PAGE, sEVs lysates were denatured by incubation with sample buffer at 95°C for 10 min. SDS-PAGE-separated protein samples were electro-transferred to polyvinylidene fluoride membrane (Beyotime, China) using wet transfer (BG-blotMINI, China). The membrane was immunoblotted with 1 mg/ml primary Abs, followed by a respective secondary HRP-labeled anti-IgG (Beyotime, China). The protein bands were visualized with an ECL detection system (Amersham, USA). The antibodies for eIF2α and phospho-eIF2α West blotting were purchased from Cell Signaling technology, Inc., USA. The clone EPR7130 (B) (abcam, UK) were used for detecting TSG101. The clone EPR23105-125 (abcam, UK) were used for detecting CD9.

### Statistical analyses

Data are expressed as means ± SD. Student’s t test was used only when the two sets of data conformed to normal distribution and had the same SD, regardless of sample size. Otherwise, nonparametric tests were used. Insulitis score was analyzed by Chi square test. Statistical analyses were performed in Prism 8 (GraphPad software Inc, USA). P <0.05 was considered statistically significant.

## Results

### Sustained high-glucose intake aggravates islet inflammation and accelerates the onset of T1D in NOD mice

**Figure 1:**
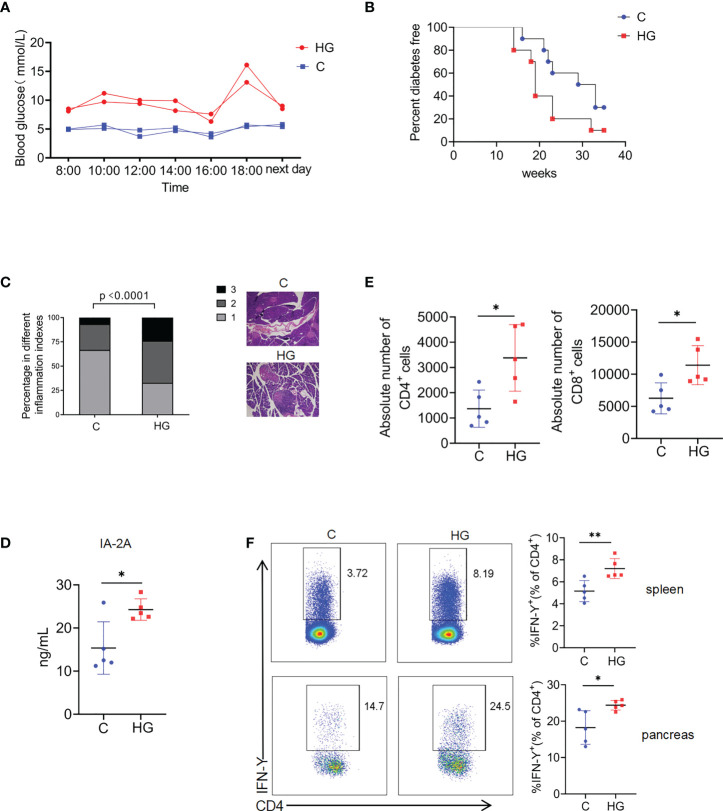
Sustained high-glucose intake aggravates islet inflammation and accelerates the onset of T1D in NOD mice **(A)** Blood glucose at different time points in a day in NOD mice given normal water **(C)** or high-glucose water (HG) (n=2). **(B)** The diabetes-free percentage in NOD mice given normal water **(C)** or high-glucose water (HG) over time, significance was determined by Gehan-Breslow-Wilcoxon test, P<0.05 (n=10). **(C)** Representative images of HE staining of pancreas (magnification 20×) and insulitis scores in NOD mice given normal water **(C)** or high-glucose water (HG) (n=5). Statistical significance was analyzed by Chi square test, p<0.0001. **(D)** Serum concentrations of Insulinoma Associated-2 Autoantibodies (IA-2A) in NOD mice given normal water **(C)** or high-glucose water (HG) (n=5). **(E)**The absolute number of pancreas-infiltrating CD4^+^ and CD8^+^ T cells of NOD mice given normal water **(C)** or high-glucose water (HG) (n=5). **(F)** The secretion of IFN-γ by CD4^+^ T cells in the spleen (up) and pancreas (down) of NOD mice given normal water **(C)** or high-glucose water (HG) (n=5). Data are representative of two independent experiments. Summary data are presented as mean ± SD. *p < 0.05, and **p < 0.01, unpaired two-tailed Student’s t tests.

We first determined whether high-glucose intake accelerates the onset of T1D in NOD mice. It was observed that dynamic blood glucose levels of NOD mice fed with high-glucose water for 1 weeks fluctuated in a relatively high range at different time points throughout the day, compared with control NOD mice fed with normal water (**Figure 1A**). Continuous blood glucose monitoring indicated that the level of blood glucose in NOD mice treated with high-glucose water were well controlled in a relatively safe range before 12 weeks of age (**Supplementary Figure 1**). As expected, control NOD mice started to develop diabetes at about 16 weeks of age, and 70% became diabetic by the age of 30 weeks. However, NOD mice fed with high-glucose drinking began to develop diabetes much earlier, and 90% became diabetic by the age of 30 weeks (**Figure 1B**). Consistently, histopathological analysis revealed that sustained high-glucose drinking aggravates the development of insulitis in NOD mice at 12 weeks of age (**Figure 1C**). We then examined the levels of autoantibodies and proinflammatory T cell responses in NOD mice fed with different drinking at 12-14 weeks of age. When compared with control NOD mice, serum levels of autoantibodies against Insulinoma associated protein 2 (IA-2), one of major T1D autoantigens, were significantly increased in the NOD mice fed with glucose drinking for 3 weeks (**Figure 1D**). As expected, the absolute number of pancreas-infiltrating CD4^+^ and CD8^+^ T cells was markedly increased in NOD mice fed with high-glucose water compared to control NOD mice (**Figure 1E**). The frequency of IFN-γ-producing CD4^+^ T cells in the spleen and pancreas of NOD mice given high-glucose drinking was significantly higher than that of NOD mice given normal drinking (**Figure 1F**). The frequency of IFN-γ-producing CD8^+^ T cells and Th17 cells in the spleen and pancreas were no significant difference between the two groups (**Supplementary Figure 2**). Together, these data indicate that sustained high-glucose drinking aggravates islet inflammation, enhances autoimmune response, and accelerates the onset of T1D in NOD mice.

### High-glucose induces up-regulation of islet beta-cell autoantigens and ERS-related genes in NIT-1 cells

**Figure 2:**
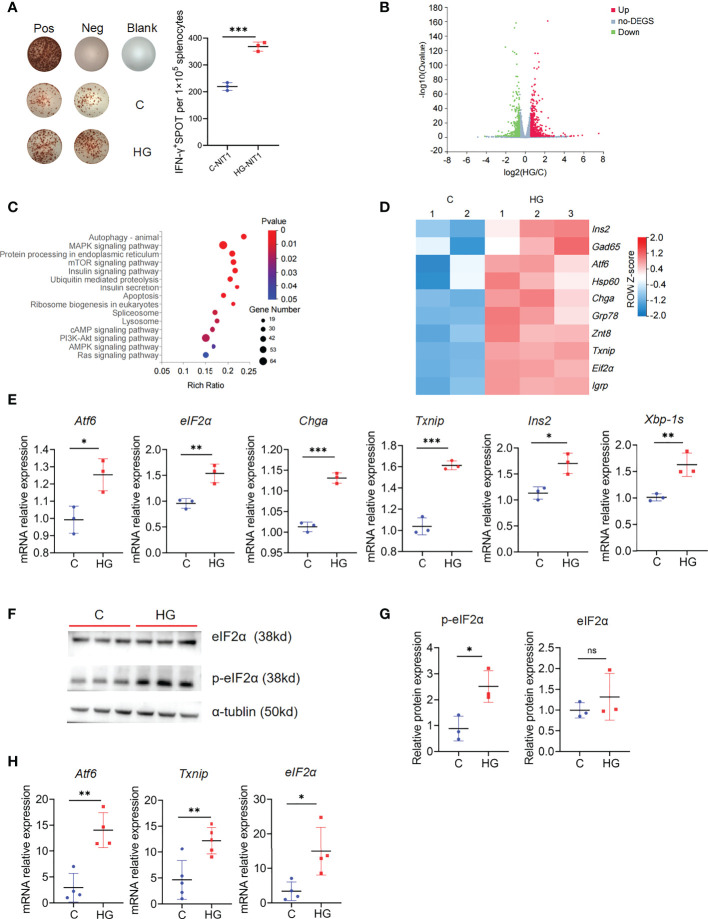
High-glucose treatment enhance the immunogenicity of NIT-1 cells and up-regulate the autoantigen and ERS related genes expression **(A)** Representative plots of IFN-γ production by NOD mouse splenocyte alone (Neg), or incubated with untreated (C-NIT1) or high-glucose treated NIT-1 cells (HG-NIT1), or PMA plus ionomycin (Pos). Summary data are presented as the mean IFN-γ spots number per 1x10^5^ splenocytes ± SD in triplicate for one representative result of at least two independent experiments. **(B)** The volcano plot of NIT-1 cell transcriptome after high-glucose treatment, 684 genes were up-regulated (red dot), and 425 genes were down-regulated (blue dot) (|FC| ≥1.5, fold change, FDR< 0.05). **(C)** KEGG pathway analysis of 684 up-regulated genes in high-glucose treated NIT-1 cell transcriptome (|FC|≥1.5, FDR<0.05). **(D)** The T1D autoantigens and ERS-related genes expression in the transcriptome of NIT-1 cell with or without high-glucose treatment, red means up-regulated and blue means down-regulated, each column depicts a biological duplication. **(E)** The mRNA levels of ERS-related genes *Atf6, eIF2α, Txnip, Chga*, *Xbp-1s* and *Ins* in NIT-1 cells with or without high-glucose treatment, which were detected by qPCR. **(F)** The protein level of p-eIF2α and eIF2α in untreated NIT-1 cell **(C)** and high-glucose treated NIT-1 cell (HG) detected by western blot. **(G)** The ratio of p-eIF2α/tublin and eIF2α/tublin were quantified. **(H)** The mRNA levels of ERS-related genes *Atf6, eIF2α* and *Txnip* in pancreatic tail tissues of NOD mouse, which were detected by qPCR (n=5). Three independent biological samples were used for quantitative PCR and WB analysis. Summary data are presented as mean ± SEM. *p < 0.05, **p < 0.01, and ***p < 0.001, unpaired two-tailed Student’s t tests.

Since islet beta cells are extremely sensitive to high-glucose exposure, then we questioned whether high-glucose treatment increased the visibility of beta cells to the immune system. IFN-γ ELISPOT analysis showed that high-glucose-treated NIT-1 cells stimulated significantly increased IFN-γ secretion in splenocytes from NOD mice compared with untreated NIT-1 cells (**Figure 2A**). To further explore the possible mechanisms, we performed global RNA-seq analysis of NIT-1 cells treated with or without high-glucose (**Supplementary Table 1**). Upon high-glucose treatment, 684 unique genes were significantly upregulated and 425 unique genes were significantly downregulated (FDR<0.05 and fold change [FC]≥1.5) in NIT-1 cells (**Figure 2B**; **Supplementary Table 2**). KEGG pathway analysis depicted significant enrichment of DEGs belonging to pathways of autophagy, insulin, mTOR, MAPK signaling and protein processing in ER, etc. (**Figure 2C**). Moreover, ERS-related genes such as *Atf6, eIF2α* and *Txnip* and islet beta-cell autoantigen genes such as *Chga, Ins2, Hsp60* and *Gad65* were significantly up-regulated in high-glucose-treated NIT-1 cells (**Figure 2D**). Consistently, real-time quantitative PCR confirmed that mRNA levels of *Atf6, eIF2α, Txnip*, *Chga*, *Ins* and *Xbp-1s* were significantly up-regulated in NIT-1 cells treated with high-glucose (**Figure 2E**). Meanwhile, the protein level of phosphorylated-eIF2α, but not total eIF2α, was also up-regulated in NIT-1 cells treated by high-glucose (**Figures 2F,G**). Moreover, the mRNA expression levels of the ERS marker *Atf6, eIF2α* and *Txnip* were significantly increased in the pancreatic tail tissues of NOD mice drinking high-glucose water (**Figure 2H**). Collectively, these data suggest that NIT-1 cells used in our study exhibit the characteristics of primary beta cells in response to high-glucose stimulation to some extent, including upregulation of ERS and autoantigen expression, which may be related to the enhanced visibility of islet beta-cells to the immune system.

### High-glucose treatment enhances the immunostimulatory activity of NIT-1 cell-derived sEVs

**Figure 3:**
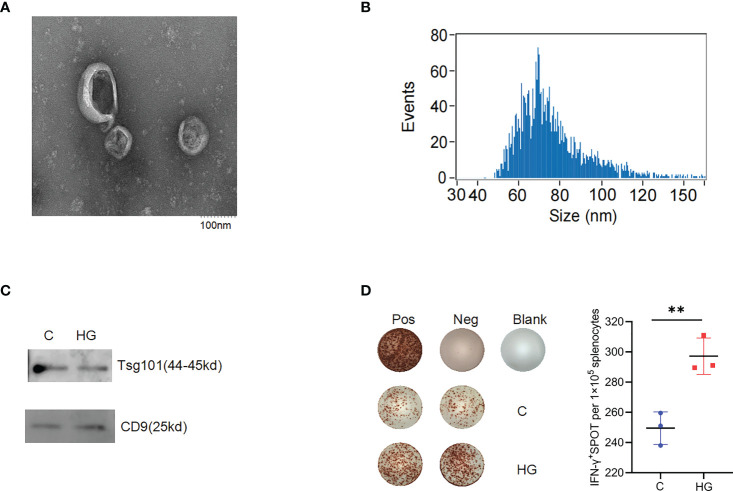
High-glucose treatment enhance the immunogenicity of sEVs derived from NIT-1 cell **(A)** The electron microscopic image of purified sEVs derived from NIT-1 cells. **(B)** The particle size distribution of sEVs derived from NIT-1 cells by Nanoparticle Tracking Analysis. **(C)** Identification of sEVs markers TSG101 and CD9 in sEVs derived from untreated NIT-1 cells **(C)** and high-glucose treated NIT-1 cells (HG) by western blot. **(D)** Representative plots of IFN-γ production by NOD mouse splenocytes alone (Neg), or incubated with PMA plus ionomycin (Pos), or sEVs derived from untreated **(C)**, or high-glucose treated (HG) NIT-1 cells. Summary data are presented as the mean IFN-γ spots number per 1x10^5^ splenocytes ± SD in triplicate for one representative result of two independent experiments. **p < 0.01, unpaired two-tailed Student’s t tests.

Since sEVs are considered as extracellular messengers with potent immune-modulatory activities linking islet beta-cells to immune cells, we next explored whether high-glucose treatment affects the immunostimulatory activity of sEVs released by ER stressed islet beta-cells. The sEVs were collected from the culture supernatants of NIT-1 cells treated with or without high-glucose through sequential ultracentrifugation. The purified sEVs particles were round vesicles about 100 nm in size, as demonstrated by the electron micrograph (**Figure 3A**). Consistently, the particle size detection results showed that most vesicles had a particle size of 30-150nm (**Figure 3B**). Meanwhile, we observed that high-glucose treatment seemed to induce more formation and release of sEVs from NIT-1 cells (**Supplementary Figure 3**). The enrichment of sEVs proteins such as tumor susceptibility gene 101 protein (TSG101) and CD9 antigen (CD9) in sEVs samples was identified by western blot analysis (**Figure 3C**). To examine the immunostimulatory activity of sEVs released by NIT-1 cells under high-glucose or normal culture condition, splenocytes from 6- to 8-week-old NOD mice were incubated with sEV samples (9×10^9^ particles/ml) for 24 h, and IFN-γ secretion were measured by ELISPOT assay. Compared with the sEVs released by NIT-1 cells under normal culture condition (C-sEV), the same dose of sEVs derived from high-glucose treated-NIT-1 cells (HG-sEV) stimulated significantly increased IFN-γ secretion in splenocytes from NOD mice (**Figure 3D**). These results suggest that high glucose-induced ERS conditions significantly enhances the immunostimulatory activity of NIT-1 cells-derived sEVs.

### High-glucose treatment alters the proteome of NIT-1 cells-derived sEVs

**Figure 4:**
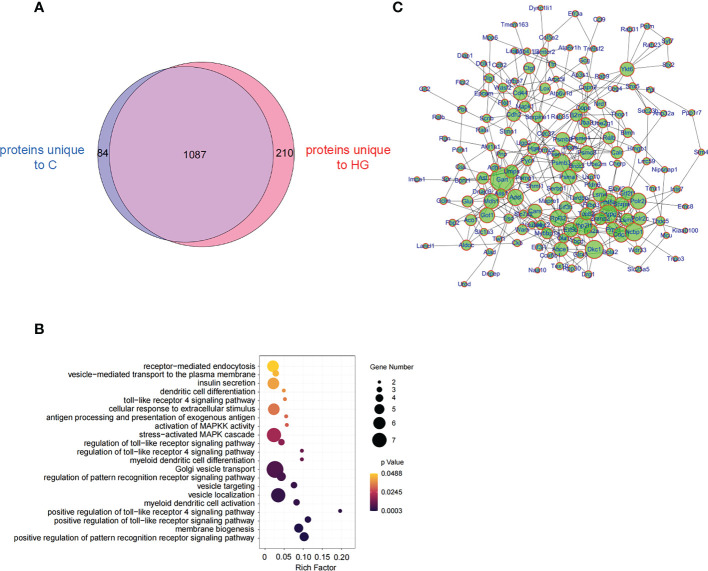
High-glucose treatment reshape the proteome of NIT-1 cell-derived sEVs **(A)** Venn diagram of proteins identified from high-glucose treated (HG) or untreated **(C)** NIT-1 cell. **(B)** GO analysis of 210 unique proteins in high-glucose treated NIT-1 cells. **(C)** A protein-protein interaction (PPI) network of unique proteins derived from high glucose treated NIT-1 cells (HG).

Then we were encouraged to study whether high glucose-induced ERS state can affect the composition of protein cargo of sEVs released by stressed islet beta-cells. Three independent sEVs samples purified from the culture supernatant of NIT-1 cells treated with or without high glucose were analyzed by LC-MS/MS. The MS data were identified using the MaxQuant’s integrated Andromeda engine (**Supplementary Table 3** for analysis parameters). A total of 1381 proteins and 6250 peptides were identified in all samples (**Supplementary Tables 4**, **5**), including several sEVs markers, such as FLOT-1, CD81, CD63, syntenin-1 and TSG101. A few of T1D-associated autoantigens, such as Chromogranin-A (ChgA), 60 kDa heat shock protein (HSP60) and the beta-cell stress marker heat shock protein 90 (HSP90), were identified in these NIT-1-released sEVs proteins. However, some major autoantigens in T1D such as Insulin, Proinsulin, GAD65, IA-2, IGRP, ZnT8 and ICA69 were not found in these sEVs proteins. A comparative analysis of proteomic data between HG-sEV and C-sEV showed that 1087 proteins were shared in common, 210 proteins were unique to HG-sEV (**Supplementary Table 6**), and 84 proteins were unique to C-sEV (**Figure 4A**). Among the shared proteins, only five proteins (ELAV1, PGBM, SL7A1, PRIO, IF2B) were significantly upregulated in HG-sEV compared to C-sEV, while three proteins (SYDC, MIF, PSB2) were significantly down-regulated, probably due to reproducibility limitations in both biological samples and mass spectrometry (**Supplementary Table 7**). So far, little is known about the direct relationship between these proteins and the pathogenesis of T1D.

We then mapped 210 HG-sEV exclusive proteins to the GO enrichment analysis and found that the significantly enriched biological process terms included toll-like receptor (TLR) signaling pathway, and pathway of DC activation and differentiation (**Figure 4B**). By analyzing the protein-protein interaction network of 210 HG-sEV proteins, we noticed that these proteins have close interactions (**Figure 4C**). These results show that high glucose-induced ERS state alters the proteome of NIT-1 cell-derived sEVs, and we presume that some HG-sEV exclusive components are probably associated with DC activation and function, which might be involved in the enhanced immunostimulatory activity of HG-sEV.

### The enhanced immunostimulatory activity of sEVs derived from high glucose treated-NIT-1 cells is largely dependent on the enhanced DC function

**Figure 5:**
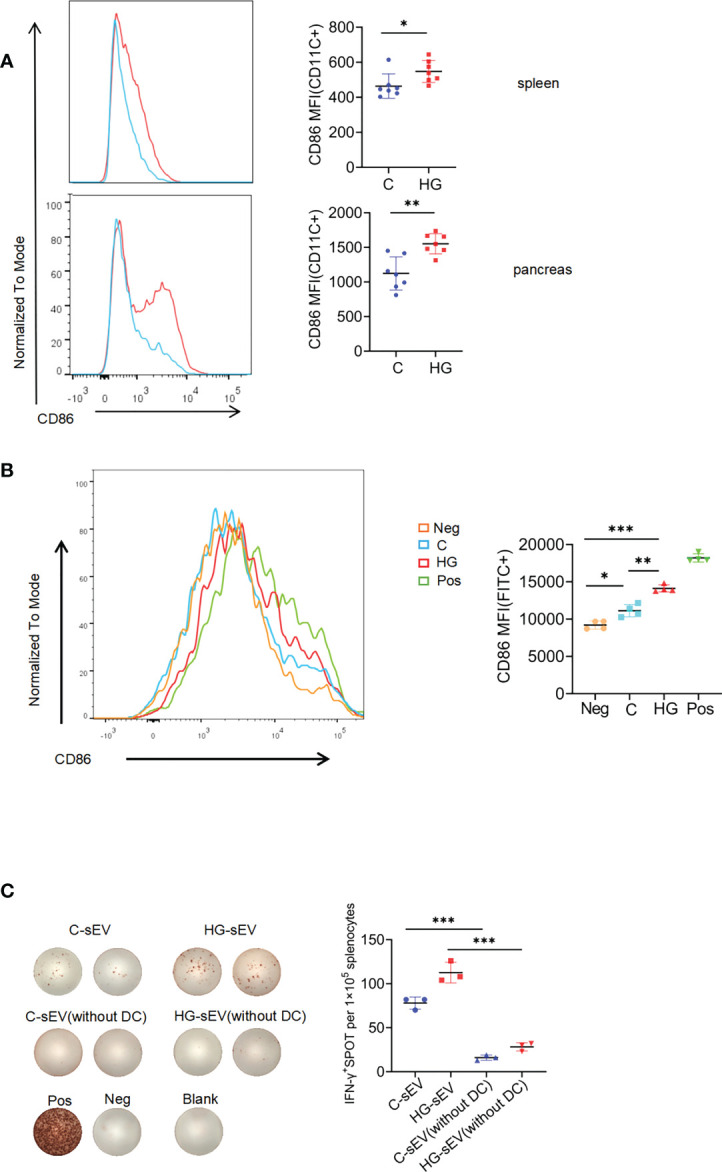
sEVs communicate with immune system *via* dendritic cells **(A)** The expression of costimulatory molecule CD86 on CD11c^+^ subset in spleen and pancreatic infiltrating cells of NOD mice given normal water **(C)** and high-glucose water (HG) (n=7). **(B)** The expression of CD86 on bone marrow-derived dendritic cells alone (Neg), or co-cultured with sEVs released from untreated **(C)**, or high-glucose treated NIT-1 cells (HG), or LPS (Pos). **(C)** Representative plots of IFN-γ production by NOD mouse splenocytes (with or without dendritic cells) alone (Neg), or incubated with untreated NIT-1 cells-derived sEVs (C-sEV) or high-glucose treated NIT-1 cells-derived sEVs (HG-sEV), or PMA plus ionomycin (Pos). Summary data are presented as mean ± SD for one representative result of two independent experiments. *p < 0.05, **p < 0.01, and ***p < 0.001, unpaired two-tailed Student’s t tests.

In the pathogenesis of T1D, DCs play a key role in antigen capture and presentation to autoreactive T cells. We observed that the expression level of costimulatory molecule CD86 on the surface of DCs in spleen and pancreas of NOD mice with continuous high-glucose drinking water was significantly higher than that in NOD mice with normal drinking water, suggesting that high-glucose treatment may promote DC maturation *in vivo* (**Figure 5A**). Since sEVs are small particles of 30 to 150nm in diameter, DCs may be more efficient at capturing particles in this size range than other subpopulations, such as macrophages or B cells. Therefore, we asked whether HG-sEV has a stronger ability to stimulate DC maturation than C-sEV. FACS analysis demonstrated that HG-sEV stimulation significantly increased the expression levels of CD86 on the bone marrow-derived DCs cultured *in vitro* compared with C-sEV stimulation (**Figure 5B**).

Finally, we wished to determine whether the enhanced immunostimulatory activity of sEVs released by NIT-1 cells with high-glucose treatment could be mediated by DC function. CD11c positive cells in mouse spleen cells were effectively removed by magnetic sorting (**Supplementary Figure 4**). The same number of whole splenocytes or CD11c^+^ cells-removed splenocytes from 6- to 8-wk-old NOD mice were incubated with C-sEV or HG-sEV (9*10^9^ particles/ml) for 24h, and IFN-γ secretion were measured by ELISPOT assay. Although HG-sEV consistently displayed a significantly enhanced ability to stimulate IFN-γ secretion by whole splenocytes compared with C-sEV, this effect was almost eliminated after the removal of CD11c^+^ cells (**Figure 5C**). Collectively, these data suggest that the enhanced immunostimulatory activity of sEVs derived from NIT-1 cells treated with high-glucose is largely dependent on enhanced DC function.

## Discussion

High total sugar intakes (20) and higher glycaemic index of the diet (24) have been thought to be associated with increased risk of progression to T1D in children. Nevertheless, there is a lack of direct evidence available that illustrates the effects of high-glucose intake on T1D progression. In the present study, we provided laboratory evidence that sustained high-glucose drinking significantly promotes the onset of T1D in NOD mice. Mechanistically, high-glucose treatment induces aberrant ERS, up-regulates the expression of autoantigens in islet beta cell, and enhances the visibility of islet beta-cells to the immune system. Moreover, high-glucose treatment alters the proteome of beta-cells-derived sEVs, and significantly enhances the ability of sEVs to promote DC maturation and stimulate autoimmune response.

Dietary sugar consumption is a controversial public health subject. Children are most attracted to drinks and foods high in sugar. For example, Coca-Cola, the most popular soft-drink in the world, contains more than 10% sugar. High sugar intake has been associated to increase the risk for obesity, T2D, cardiovascular diseases, and cancer (25–28). The incidence of T1D and T2D showed a concomitant increase, suggesting that they may share a common environmental trigger. We hypothesized that excessive intake of sugar, from various high-sugar beverages and high-glycemic index foods, can temporarily expose the body’s islet beta cells to high glucose influx and high intracellular glucose levels, which may lead to islet beta cells hyperactivity and ERS, thereby increasing the visibility of beta cells to the immune system and accelerating autoimmune destruction of beta cells in individuals genetically susceptible to T1D. To test this hypothesis, NOD mice were allowed to freely intake 1.1M (20%) high-glucose water, which is expected to maximally and safely simulate individual over-consumption of various high sugar beverages and high-glycemic index foods, ultimately leading to rapid absorption of a large amount of glucose into the blood. Other studies have used such high concentration sugar drinking water as the only source of water for mice. For example, EAE model mouse was treated with 20% high-glucose water to study the role of high-glucose intake in promoting autoimmunity and inflammation (29). In another study on how fructose triggers hepatosteatosis and NASH, mice were allowed free access to 30% fructose in water solution for more than 10 months (30). The published studies have shown that long-term intake of high sugar drinking water has no obvious toxic effect. It was shown that high fructose and sucrose water intake for 10 weeks don’t cause significant change of serum uric acid that is one of indexes of kidney injury (31). A study about the long-term effects of mannose (C-2 epimer of glucose) supplementation indicated that intake of 20% mannose in the drinking water for 5 months does not cause bloating, diarrhea, abnormal behavior, weight change, increase in hemoglobin glycation, or histology changes of organs (32).

Our study proved that NOD mice given high-glucose water exhibited significantly earlier onset of T1D and more severe autoimmunity-mediated islet inflammation. We found that free intake of high-glucose water kept the daily dynamic random blood glucose of NOD mice at a relatively and temporarily high level, but has not yet met the diagnostic criteria for diabetes, that is, the random blood glucose value greater than 13.9 mM for 2 consecutive days. In response to this irregular but transient significant increase in blood glucose, beta cells need to produce more insulin to control blood glucose in a relatively safe range, resulting in an imbalance in ER protein load and ER folding capacity, known as ERS. With the gradual destruction of islet beta cells, NOD mice could no longer maintain normal blood glucose level and then developed T1D. In T2D, dysregulated ERS occurs in beta cells exposed to high blood glucose, leading to beta cell dysfunction and apoptosis (28). As a possible mechanism for the induction of autoimmunity in T1D, abnormal ERS may result in the generation of autoantigens by post-translational modification of proteins in beta cells (8, 33–35). Our study confirmed that the expression levels of genes related to ERS and autoantigens in NIT-1 beta cells were significantly increased under high-glucose treatment, and more importantly, the ability of NIT-1 beta cells to stimulate IFN-γ secretion by splenocytes from NOD mice was significantly enhanced by high-glucose treatment. This is consistent with previous studies showing that the ability of beta cells to produce autoantigens and activate autoreactive T cells was enhanced under high-glucose treatment (36, 37). In fact, it has been suggested that NIT-1 beta cell line is a useful tool to understand changes in primary islet beta cells in the pathogenesis of T1D (38, 39). We also confirmed that free intake of high-glucose water can induce the up-regulation of ERS markers, such as *Txnip, eIF2α, Atf6* in the pancreatic tissues of NOD mice, accompanied by enhanced levels of autoantibodies and IFN-γ producing T cell responses. Increasing levels of IFN-γ have been shown to correlate with diabetes progression in NOD mice as well as being necessary for virus-related diabetes (40, 41). Although it has been shown that high-glucose intake exacerbates autoimmunity in experimental colitis and experimental autoimmune encephalomyelitis by promoting Th17 cell differentiation (30), we did not observe a significant increase in Th17 cells in the spleen and pancreas of NOD mice fed with high-glucose water.

DCs have been shown to be essential in T cell-mediated destruction of beta cells in T1D by making beta cells visible to the immune system through uptake, processing and presentation of beta cells-derived autoantigens (42–44). Recently, sEVs from beta cells or mesenchymal stem cells have emerged as novel autoimmune targets, carrying autoantigens and other molecules that can stimulate both innate and adaptive immune responses in T1D (11, 45–51). Researchers found that beta cell autoantigens are taken up, processed and presented by DCs *via* sEVs, thereby initiating autoreactive T cell responses and promoting T1D development (52). Since it’s very difficult to get enough sEVs from primary islet beta cells, several insulinoma cells such as NIT-1, MIN-6, INS-1, etc. have been used to isolate sEVs and to study the role of sEVs in the pathogenesis of T1D due to their low experimental cost and better availability (46, 47, 53). A proteomics study of sEVs derived from MIN-6 cells suggested that intercellular communication mediated by sEVs transfer among tissues may account for the major reason of beta cell destruction (54). Our data showed that high-glucose treated NIT-1 cells tend to release more sEVs than untreated NIT-1 cells. Consistently, high glucose-stimulated human mesangial cells released a higher number of sEVs compared to unstimulated cells (55). Similar effects were also observed in trophoblastic cells (56). Thus, the effect of high-glucose on sEVs secretion deserves more attention. Notably, purified sEVs secreted from NIT-1 cells effectively stimulates splenocytes from NOD mice to secrete inflammatory cytokines, and high-glucose treatment significantly enhances the immunostimulatory activity of NIT-1 cells-derived sEVs. And removal of CD11c-positive cells from splenocytes of NOD mice dramatically reduced the ability of sEVs to stimulate IFN-γ secretion by splenocytes. Considering that CD11c-positive cell subset represents the majority of antigen-presenting cells (APC), since CD11c is expressed on DCs, monocytes, macrophages and a subset of B cells, and that CD11c-positive classical DCs can capture sEVs more efficiently than macrophages and B cells (57), we hypothesized that this effect might be attributed to enhanced DC antigen presentation function mediated by sEVs.

Given that different types of cellular stress affect the proteome composition of sEVs (58), and stress-induced exosomal release of intracellular autoantigens and immunostimulatory chaperones may play a role in the initiation of autoimmune responses in T1D (11), we speculate that high glucose-induced ERS might be related to change in sEVs profile that enhances autoantigenicity in T1D. Our proteomics study identified much more proteins in NIT-1 cell-derived sEVs than a previous study by Lee et al. (53), and most proteins (181 in 269) identified in Lee’s study were also found by our study. We found that high-glucose treatment greatly altered the proteome of NIT-1 cell-derived sEVs. However, only limited autoantigens, such as ChgA and HSP60, were identified in these NIT-1-released sEVs proteins, but their content in sEVs were not changed by high-glucose treatment. Several common beta cell autoantigens, such as Insulin, GAD65, IA-2, ZnT8, were not found in NIT-1-released sEVs proteins. Consistently, Lee et al. also failed to identify these known autoantigens in the NIT-1-derived sEVs proteome (53). There are several possible explanations for this: 1. the abundance of these known autoantigens contained in NIT-1-derived sEVs may be lower than the detection range of mass spectrometry; 2. Some known autoantigens contained in NIT-1-derived sEVs may exist as peptides segments rather than complete proteins. If these peptides are further degraded by trypsin during sample preparation into short peptides less than 6 amino acids in length, they will be directly filtered out during data analysis; 3. The sEVs may contain certain unconventional autoantigens with strong immunogenicity, which are encoded by noncoding RNA or unconventional open reading frame and cannot be identified by traditional proteomics. More studies are needed to confirm these speculations.

There is another possibility that high-glucose treatment may further enhance the ability of sEVs to promote CD11c^+^DC maturation, providing a stronger costimulatory signal for the activation of autoreactive T cells, which may ultimately induce stronger autoreactive T cell activation. Our MS data preliminarily revealed that a series of proteins exclusively present in HG-sEV are enriched in DC differentiation, activation, and function-related pathways.Consistent with this finding, HG-sEV promoted the expression of the costimulatory molecule CD86 on the surface of bone-marrow derived DCs compared with C-sEV. Thus, we speculate that some components (e.g., proteins) in HG-sEV might enhance the immunostimulatory activity of HG-sEV by modulating the activation and function of DC. However, the molecular mechanisms underlying this process needs to be further studied.

There are several limitations in this study. First, this study lacked a ‘low glucose’ control group, which could have served as control for not only the total amount of consumption but the actual concentration of sugar. Second, there is a bit of a leap between using the NIT-1 beta cell line and presuming the role of primary NOD islet beta cells *in vivo* during T1D, since there are some differences between this transformed cell line and primary NOD beta cells. Third, this study did not investigate the specific components and underlying mechanisms of high-glucose treated-NIT-1 beta cell-derived sEVs that enhance DC function.

Taken together, we provide evidence for negative effect of high-glucose intake as a dietary factor on the pathogenesis of T1D. Our findings suggest that sustained high-glucose intake leads to abnormal ERS in islet beta cells of NOD mice, which enhances the visibility of islet beta cells to the immune system. Meanwhile, high-glucose treatment may increase the ability of islet beta cells-derived sEVs to promote CD11c^+^ DC maturation and antigen presentation, ultimately leading to an enhanced T-cell response to beta cells in NOD mice. Therefore, avoiding high sugar intake may be an effective disease prevention strategy for children or adults susceptible to T1D.

## Data availability statement

The data presented in the study are deposited in the ProteomeXchange repository, accession number PXD032768 and Sequence Read Archive (SRA) repository, accession number PRJNA816263.

## Ethics statement

The animal study was reviewed and approved by Animal Ethics Committee of the Army Medical University (Third Military Medical University).

## Author contributions

XL and LNW performed main experiments and data analysis, and draft the manuscript. GM, XC prepared the biological samples and performed histopathological analysis. SY, MZ, ZZ and JZ isolated and identified sEVs. ZL analyzed MS data. YW interpreted the results and evaluated the data. LW supervised and managed the research process, completed the manuscript and provided research funds. All authors contributed to the article and approved the submitted version.

## Funding

This work was supported by the grant from National Natural Science Foundation of China [grant numbers 82071825 and 81871301] awarded to LW.

## Acknowledgments

We would like to acknowledge BGI Genomics Co., Ltd for their assistance in RNAseq and proteome bioinformatics analysis.

## Conflict of interest

The authors declare that the research was conducted in the absence of any commercial or financial relationships that could be construed as a potential conflict of interest.

## Publisher’s note

All claims expressed in this article are solely those of the authors and do not necessarily represent those of their affiliated organizations, or those of the publisher, the editors and the reviewers. Any product that may be evaluated in this article, or claim that may be made by its manufacturer, is not guaranteed or endorsed by the publisher.

## Abbreviations

ER, Endoplasmic reticulum; SSBs, Sugar-sweetened beverages; DEGs, Differentially expressed genes; GO, Gene Ontology; KEGG, Kyoto Encyclopedia of Genes and Genomes; ELISPOT, Enzyme-linked Immunospot; IA-2, Insulinoma-associated protein 2; DC, Dendritic cell; APC, Antigen-presenting cell; sEVs, small Extracellular Vesicles; eIF-2α, eukaryotic initiation factor-2α; The mass spectrometry proteomics data have been deposited to the ProteomeXchange Consortium *via* the PRIDE partner repository with the dataset identifier PXD032768.; RNAseq data of NIT-1 cells from control and high glucose group have been uploaded to Sequence Read Archive (SRA) with BioProject ID: PRJNA816263.
