# Supplementary figures and images for: Sustained high glucose intake accelerates type 1 diabetes in NOD mice
Source: Front Endocrinol (Lausanne). 2022 Dec 5;13:1037822. doi: 10.3389/fendo.2022.1037822 (PMC9760976; doi:10.3389/fendo.2022.1037822)

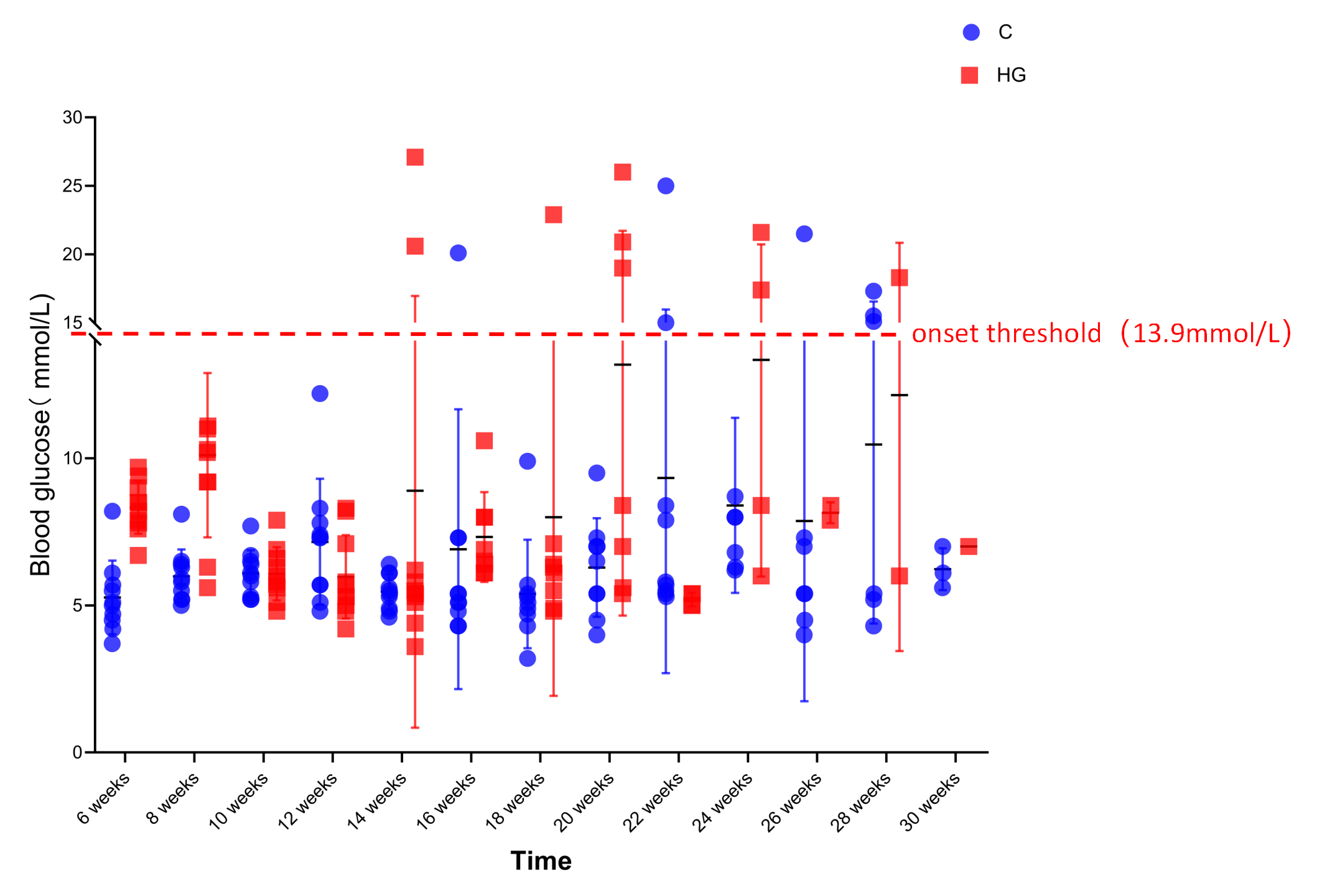

Supplement: Supplementary file 1 [file Image_1.tif]

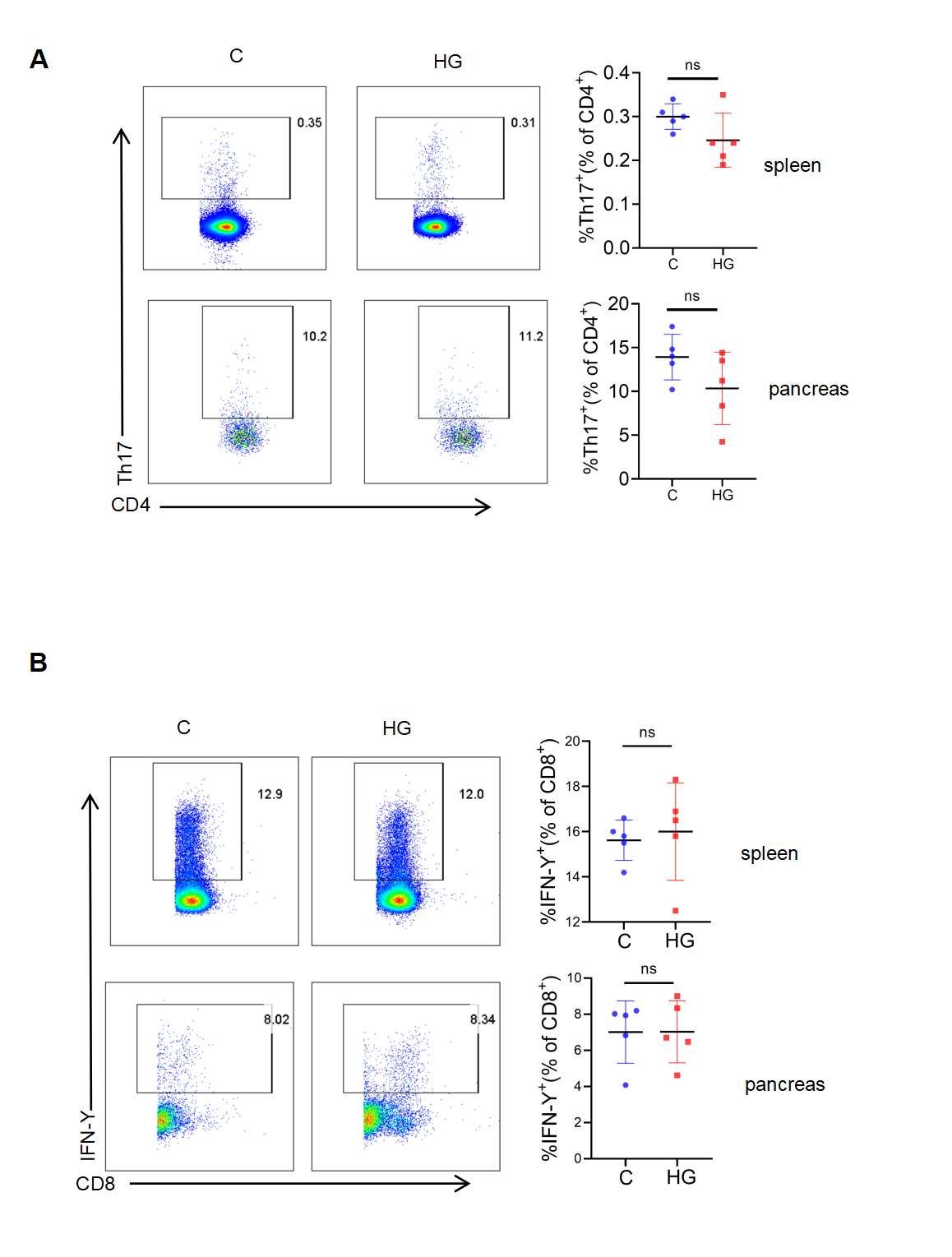

Supplement: Supplementary file 2 [file Image_2.tif]

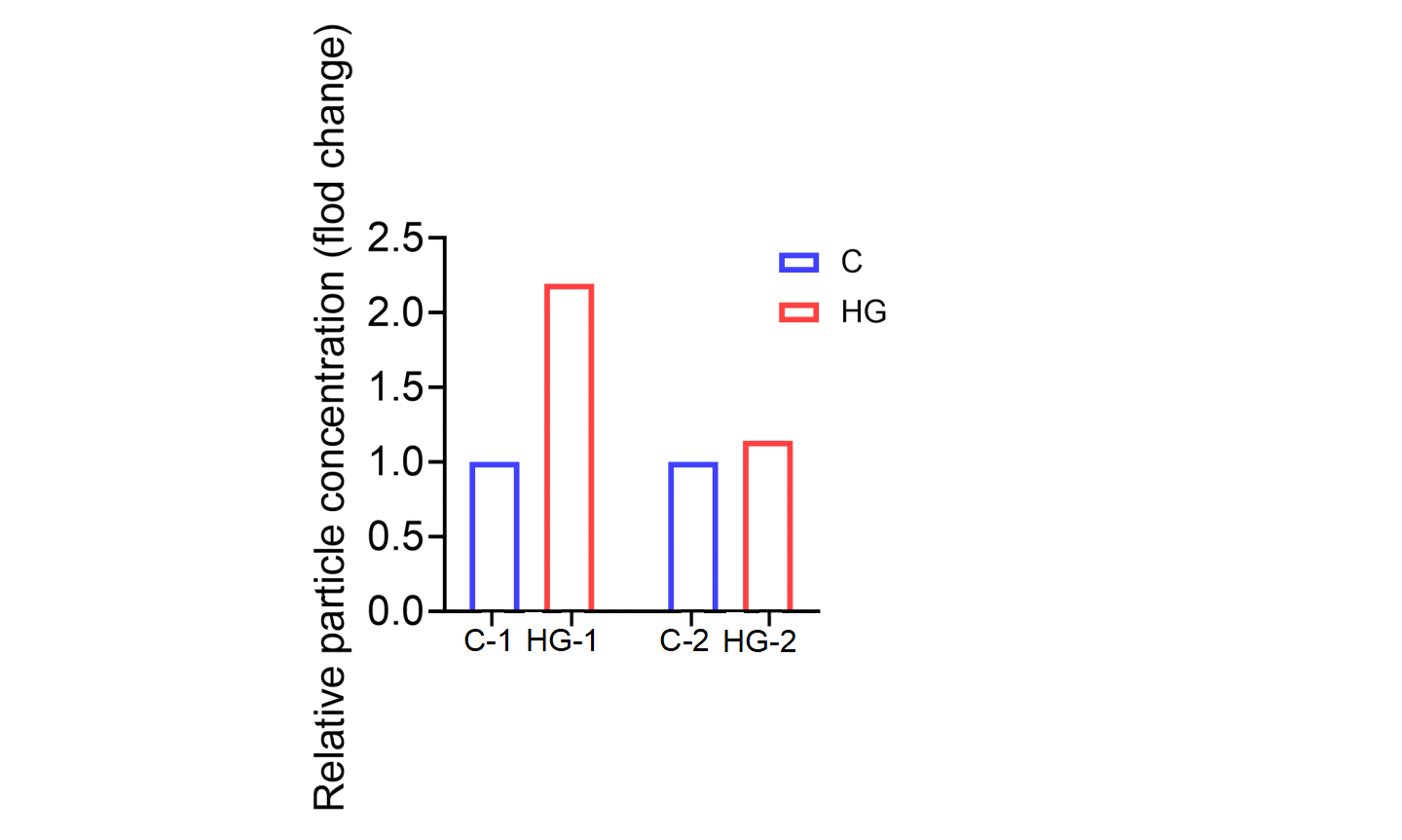

Supplement: Supplementary file 3 [file Image_3.tif]

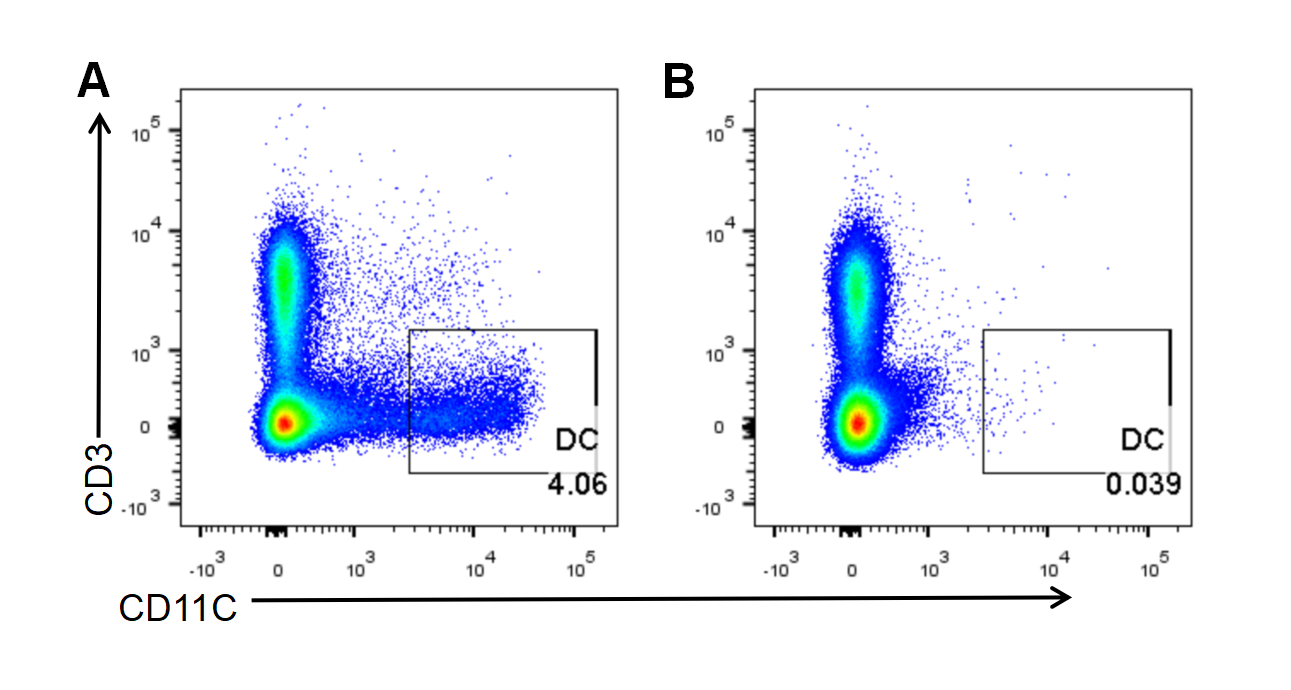

Supplement: Supplementary file 4 [file Image_4.tif]
